# Prognostic value for mortality of the new FADOI-COMPLIMED score(s) in patients hospitalized in medical wards

**DOI:** 10.1371/journal.pone.0219767

**Published:** 2019-07-24

**Authors:** Roberto Nardi, Carlo Nozzoli, Franco Berti, Erminio Bonizzoni, Leonardo M. Fabbri, Stefania Frasson, Maurizia Gambacorta, Marilisa Martini, Antonino Mazzone, Carlo Lorenzo Muzzulini, Alessandro Nobili, Mauro Campanini

**Affiliations:** 1 Internal Medicine, “Maggiore” Hospital, Bologna, Italy; 2 Department of Internal Medicine, Careggi Hospital, Florence, Italy; 3 Internal Medicine, San Camillo Forlanini Hospital, Rome, Italy; 4 Institute Department of Clinical Sciences and Community, Section of Medical Statistics, Biometry and Epidemiology, Faculty of Medicine and Surgery, University of Milan, Milan, Italy; 5 Department of Internal and Respiratory Medicine, University of Modena & Reggio Emilia, Modena, Italy; 6 Research Department FADOI Foundation, Milan, Italy; 7 Internal Medicine, Hospital Media Valle del Tevere, Todi, Italy; 8 Internal Medicine, San Bortolo Hospital, Vicenza, Italy; 9 Department of Internal Medicine, Civile Hospital, Legnano, Italy; 10 Internal Medicine, Ceva Hospital, Ceva, Italy; 11 Laboratory for Quality Assessment of Geriatric Therapies and Services, Department of Neuroscience, IRCCS- Istituto di Ricerche Farmacologiche Mario Negri, Milan, Italy; 12 Department of Internal Medicine, Hospital ‘Maggiore della Carità’, Novara, Italy; Universidad Miguel Hernandez de Elche, SPAIN

## Abstract

**Background:**

Recently we defined a user-friendly tool (FADOI-COMPLIMED scores—FCS) to assess complexity of patients hospitalized in medical wards. FCS-1 is an average between the Barthel Index and the Exton-Smith score, while FCS-2 is obtained by using the Charlson score. The aim of this paper is to assess the ability of the FCS to predict mortality in-hospital and after 1-3-6-12-months. In this perspective, we performed comparisons with the validated Multidimensional Prognostic Index (MPI).

**Methods:**

It is a multicenter, prospective observational study, enrolling patients aged over 40, suffering from at least two chronic diseases and consecutively admitted to Internal Medicine departments. For each patient, data from 13 questionnaires were collected. Survival follow-up was conducted at 1-3-6-12 months after discharge. The relationships between cumulative incidences of death with FCS were investigated with logistic regression analyses. ROC curve analyses were performed in order to compare the predictiveness of the logistic models based on FCS with respect to those with MPI taken as reference.

**Results:**

A cohort of 541 patients was evaluated. A 10-point higher value for FCS-1 and FCS-2 leads to an increased risk of 1-year death equal to 25.0% and 27.1%, respectively. In case of in-hospital mortality, the relevant percentages were 63.1% and 15.3%. The logistic model based on FCS is significantly more predictive than the model based on MPI (which requires an almost doubled number of items) for all the time-points considered.

**Conclusions:**

Assessment of prognosis of patients has the potential to guide clinical decision-making and lead to better care. We propose a new, efficient and easy-to-use instrument based on FCS, which demonstrated a good predictive power for mortality in patients hospitalized in medical wards. This tool may be of interest for clinical practice, since it well balances feasibility (requiring the compilation of 34 items, taking around 10 minutes) and performance.

## 1. Introduction

Assessment of the prognosis of patients hospitalized in medical wards, although a challenging task, has the potential to guide clinical decision-making and to lead to better care [1]. This objective is even more critical nowadays, since a demographic evolution in recent decades has resulted in a significant rise in the number of elderly patients with multiple diseases [2,3].

Patients hospitalized in medical wards present with ever greater complexities, being extremely heterogeneous in terms of comorbidity and illness severity, functional and cognitive status, personal priorities and preferences, and social support [4,5]. There are a few prognostic indices available for hospitalised older adults. In these indices, mortality was preferably chosen as the primary outcome, since it is dichotomous and unequivocal. However, the use of these tools has not been incorporated into routine medical practice, for a number of reasons [6]. Some existing models are applicable only to specific patient populations and therefore largely inappropriate for patients with multiple diseases [7,8], or they are based on subjective risk assessment by clinicians [9]. Others require the use of lengthy formulae or knowledge of information on the functional status, which are not always available in patient records [10–13]. Yourman et al. reviewed 16 prognostic indices for older adults in a variety of clinical settings (community healthcare, care homes and hospitals). Although several high-quality tools were identified, none of them was found free from potential bias, and the authors conclude that there is still insufficient evidence to recommend their widespread use in clinical practice [1]. Finally, during recent decades new diagnostic and therapeutic options have significantly changed the clinical scenario, and concern has been raised that some indices may have lost at least part of their accuracy [14].

Recently our group has defined a new, simple and user-friendly tool (FADOI-COMPLIMED Scores) for the assessment of complexity of patients hospitalized in medical wards [15]. In this paper, we assess the ability of FADOI-COMPLIMED Scores to predict in-hospital mortality and after 1-month, 3-month, 6-month and 1-year follow-up. In order to achieve this aim, we performed comparisons with the Multidimensional Prognostic Index (MPI) used as the reference predictive model. We considered the MPI since it was developed and validated in elderly patients [16] and its use is progressively increasing; moreover, the MPI demonstrated a higher predictive power than other measurements in older hospitalised patients [17].

## 2. Materials and methods

### 2.1 Ethics approval and consent to participate

The study was conducted following Good Clinical Practice guidelines and the Declaration of Helsinki. According to Italian law, a preliminary approval for the study was obtained by the Ethics Committee of the coordinating center—Comitato Etico (CE) Milano Area C—A.O. Niguarda Ca’ Granda, Milan, and after that all the Ethics Committees of the participating centers gave their approval (Ethics Committe Provinciale Crotone, Ethics Committee Regionale Friuli Venezia-Giulia, Ethics Committee Hospital "San Donato" Arezzo, Ethics Committee Regionale Marche, Ethics Committee IRCCS "Casa Sollievo della Sofferenza" S. Giovanni Rotondo (FG), Ethics Committee ASL Roma G, Ethics Committee Legnano, Ethics Committee Hospital “Cardarelli” Napoli, Ethics Committee Hospital “Maggiore della Carità” Novara, Ethics Committte Hospital "S. Croce e Carle" Cuneo, Ethics Committee Hospital "Bianchi-Melacrino-Morelli" Reggio Calabria, Ethics Committee Provinciale Vicenza, Ethics Committee Provinciale La Spezia, Ethics Committee Hospital "S. Camillo Forlanini" Roma, Ethics Committee Provincia Agrigento, Ethics Committee AUSL Bologna, Ethics Committee Hospital "Miulli" Acquaviva nelle Fonti, Ethics Committee "ARNAS Garibaldi" Catania, Ethics Committee Hospital "Mauriziano" Torino, Ethics Committee Provinciale Savona, Ethics Committee Regionale Umbria, Ethics Committee Hospital "Careggi" Firenze, Ethics Committee “Hospital dei Colli” Napoli). Written informed consent was obtained from each participating patient.

### 2.2 Study participants, procedures and design

Patients aged over 40, suffering from at least two chronic diseases and consecutively admitted to 29 Internal Medicine (IM) departments in Italy (see complete list in the Appendix) during the period June-October 2014 were considered. For each patient, general information was collected at the time of admission to hospital (demography, routine laboratory tests, social environment / support, diseases, drug therapy). Information from a total of thirteen questionnaires was recorded for each patient. These questionnaires were chosen to evaluate domains considered representative of the complexity of patients—comorbidity (Charlson, CIRS), clinical stability (MEWS), social frailty (Flugelman), cognitive dysfunction (SPSMQ), depression (5-item GDS), functional dependence (ADL, IADL, Barthel), pressure sores (Exton-Smith scale), nutrition (MNA), pain (NRPS), and adherence to therapy (Morisky scale). As an additional criterion, the questionnaires and information to be considered for the MPI score were included, with a view to comparing the new tool we were creating with MPI as a reference. Specific training was provided to researchers in order to optimize the administration of the questionnaires and collection of data. Follow-up was conducted by telephone or by means of clinical visit at 1, 3, 6 and 12 months after discharge, to evaluate survival.

### 2.3 Rationale for FADOI-COMPLIMED score(s)

**Fig 1:**
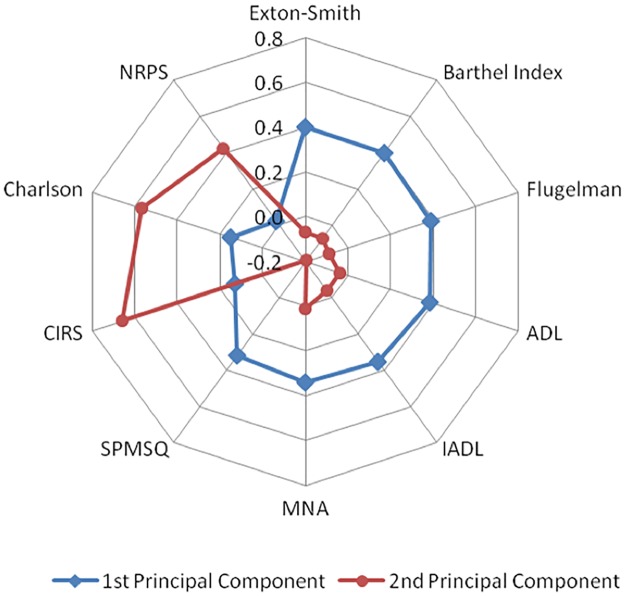
Radar plot displaying the coefficients of 1^st^ and 2^nd^ principal components derived by means of the principal component analysis. The values shown in the Radar Plot correspond to the weight (importance) assigned to each questionnaire in the calculation of the two principal components [15].

The process for derivation of the COMPLIMED score(s) has been described in a previous paper [15] and it is summarised here below. In details, a two-stage strategy was carried out consisting of a hierarchical cluster analysis followed by a principal component analysis (PCA) in order to identify and measure complexity domain(s). These multivariate analyses detected two main clusters: the first includes 7 questionnaires whose common denominator was dependence and frailty (Flugelman, SPSMQ, ADL, IADL, Barthel, Exton-Smith and MNA), the second consists of 3 questionnaires representative of comorbidity (Charlson, CIRS and NRPS). Final results of the two-stage strategy are summarized through the radar chart reported in Fig 1. The derived scores, which are expression of the two identified clusters and which are referred to as the first and second “principal components”, globally accounted for about 70% of the total information (55.2% and 13.8%, respectively). These composite scores can be further simplified in “FADOI-COMPLIMED Score 1” as a recalibrated and standardized average between the Barthel Index (BI) and the Exton Smith score (ES), and in “FADOI-COMPLIMED Score 2”, by using the Charlson score (CS) only. Therefore, the FADOI-COMPLIMED Scores only need three questionnaires to be filled out, with a total of 34 items, and can be computed through elementary steps as shown below:

1. FADOI-COMPLIMED Score 1 = mean of *BI’* and *ES’*
2. FADOI-COMPLIMED Score 2 = *CS’*

Where: *BI’* = 100–BI; *ES’* = [(20-ES)/(20–5)]x100; if CS ≤14 then CS’ = (CS/14)x100 else if CS >14 then CS’ = 100. In this way values range from 0 (best—low complexity) to 100 (worst—high complexity). Further details on FADOI-COMPLIMED Scores, and their role in assessing complexity have been reported elsewhere [15].

### 2.4 Statistical methods

As a post-hoc calculation, a total sample size of 541 subjects achieves 80% power to detect a difference of 0.07 between a “reference” predictive tool with an area under the ROC curve (AUC) of 0.70 and a “test” predictive tool with an AUC of 0.77 (i.e. a 10% increase) using a two-sided z-test at a significance level of 0.05. The predictive scores are continuous responses. The AUC was computed between false positive rates of 0.00 and 1.00, assuming a cumulative 1-year death incidence (primary outcome) equal to 35% and hypothesizing a correlation between the two predictive tools equal to 0.5 (ballpark estimate). Computations were performed using the ROC module of PASS 14 Software.

The relationships between cumulative incidences of death (in-hospital, 1-month, 3-month, 6-month and 1-year mortality) with FADOI-COMPLIMED Scores were investigated with logistic regression analyses, and results were reported as odds ratios with associated two-sided 95% CIs and two-sided p-values.

ROC curve analyses were performed in order to compare the predictiveness of the logistic models based on FADOI-COMPLIMED Scores (COMPLIMED Score Model) with respect to logistic models with MPI as covariate taken as reference. Differences between predictive models regarding the areas under the ROC curves (C-Index) were reported together with associated 95% confidence intervals (CIs) and tested for their statistical significance using the DeLong Clarke-Pearson approach. Because the C-Index is prone to a type of bias named “optimism”, we performed an internal bootstrap validation to test model adequacy and quantify “overfitting” [18]. Two sets of resampling C-Index were calculated when the logistic models were fitted on 1000 bootstrap replications (training sample) and when the retrieved model parameter estimates were evaluated against the original data (test sample). The differences between the C-Index on the bootstrap sample (training set) and the original sample (test set) were computed and averaged in order to estimate a measurement of optimism in the model fit, where optimism is the portion of predictiveness ascribed to overfitting. Finally, a corrected index was calculated by subtracting the average of the optimism estimates from the original C-Index.

All statistical computations were performed using SAS software version 9.4 and R software version 3.2.5.

## 3. Results

### 3.1 Baseline characteristics and mortality rate

A cohort of 541 consecutive patients was evaluated in the study. General characteristics of the study cohort are reported in Table 1. Mortality rates were 6.7%, 15.2%, 22.2%, 28.5% and 36.2% during hospital stay and at 1, 3, 6 and 12-month follow-up, respectively.

**Table 1:** Baseline characteristics of patients (n = 541). Values are expressed as median with Interquartile range or percentages.

Female	51%
Age (years)	79.9 (12)
< 75 years	29.6%
75–84 years	43.4%
> 85 years	27.0%
BMI	25.1 (5.9)
Underweight (<18.5)	5.8%
Normal weight (18.5–24.9)	42.6%
Overweight (> 25)	51.6%
**Chronic diseases**	
Heart failure	35.6%
Chronic obstructive pulmonary disease	35.6%
Diabetes	33.0%
Moderate/severe renal insufficiency	28.4%
Cancer	18.4%
Moderate/severe liver insufficiency	9.4%
**Number of drugs**	
at home	6 (4)
on admission to Internal Medicine	6 (5)
Caregiver YES	67.4%
Nursing home residents	2.6%
FADOI-COMPLIMED Score 1	33.3 (65.8)
< 33	49.0%
33–66	20.3%
> 66	30.7%
FADOI-COMPLIMED Score 2	28.6 (28.6)
< 33	64.9%
33–66	30.7%
> 66	4.4%
MPI Score	0.50 (0.44)
Risk Strata 1	33.5%
Risk Strata 2	35.5%
Risk Strata 3	31.0%

### 3.2 Predictiveness for mortality of FADOI-COMPLIMED scores

**Fig 2:**
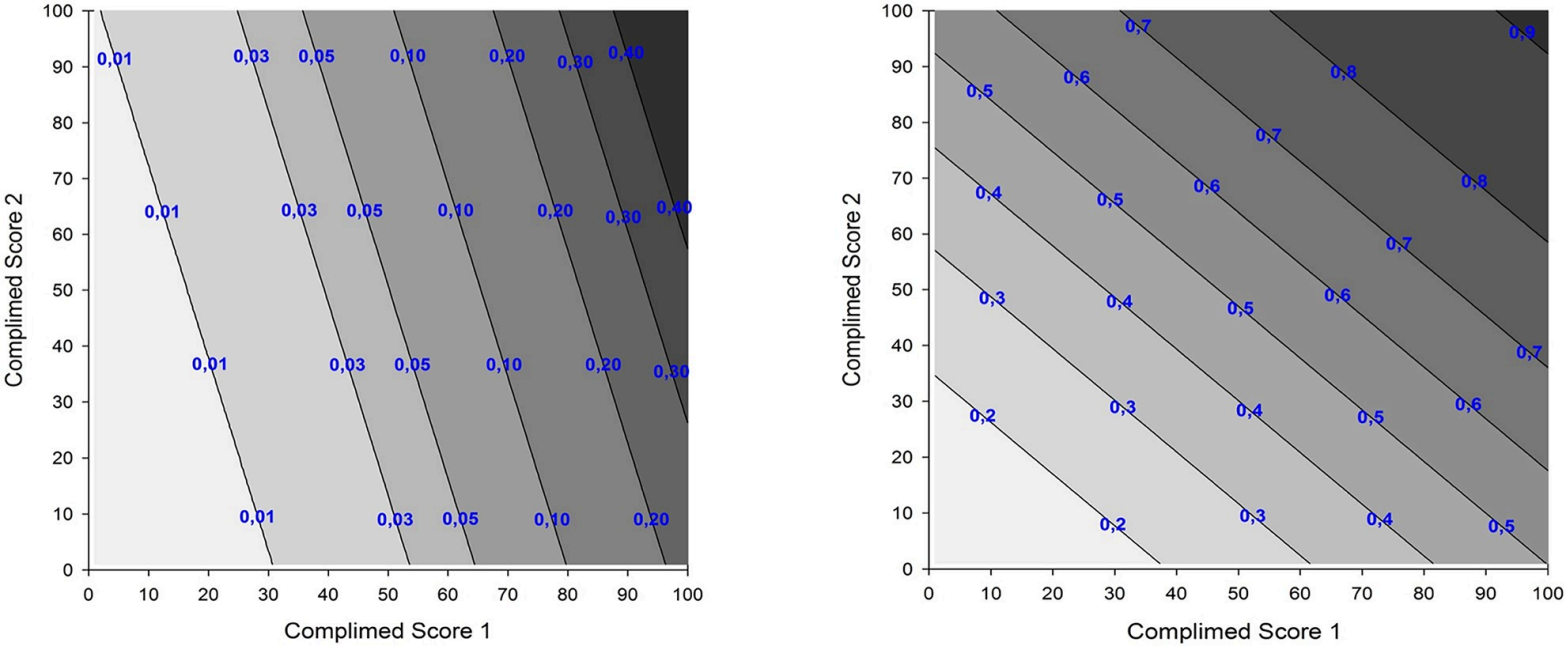
Surface plot showing the probability of in-hospital mortality (Fig 2a) and 1-year mortality (Fig 2b) as a function of FADOI-COMPLIMED score(s).

Table 2 shows the results of bivariable logistic regression analyses with FADOI-COMPLIMED Scores as prognostic factors for in-hospital, 1-month, 3-month, 6-month and 1-year cumulative mortality. According to these results, a 10-point higher value for the FADOI-COMPLIMED score 1 and the FADOI-COMPLIMED score 2 leads to an increased risk of death at 1 year equal to 25.0% and 27.1%, respectively. As far as in-hospital mortality is concerned, a clear difference exists between the contribution of dependence / frailty and that of comorbidity (the increased risk of death associated with an increase of 10 units in FADOI-COMPLIMED scores is 63.1% and 15.3%, respectively). Considering the other time-points (from 1 month to 1 year) the contribution to the risk of death of the two domains is more similar, with a trend towards a decreased relative weight for dependence / frailty and an increased contribution for comorbidity. Surface plots reported in Fig 2 with sub-figures and based on results of bivariable logistic regression analyses, show the probability gradient of in-hospital and 1-year cumulative mortality according to the combination of predefined intervals of values for FADOI-COMPLIMED Score 1 and Score 2 (surface plots for 1-month, 3-month and 6-month cumulative mortality are included in Supplementary Materials).

**Table 2:** Bivariable logistic regressions with FADOI-COMPLIMED scores as covariates. Odds ratios show the risk associated with an increase of 10 units in FADOI-COMPLIMED scores.

Clinical Event / Risk factors	Odds Ratio	95% Confidence Interval	P-Value
In-hospital Mortality			
FADOI-COMPLIMED Score 1	1.63	(1.39–1.99)	< .0001
FADOI-COMPLIMED Score 2	1.15	(0.97–1.37)	0.1093
1-month Mortality			
FADOI-COMPLIMED Score 1	1.38	(1.26–1.52)	< .0001
FADOI-COMPLIMED Score 2	1.27	(1.12–1.45)	0.0003
3-month Mortality			
FADOI-COMPLIMED Score 1	1.32	(1.23–1.42)	< .0001
FADOI-COMPLIMED Score 2	1.23	(1.09–1.38)	0.0005
6-month Mortality			
FADOI-COMPLIMED Score 1	1.31	(1.23–1.40)	< .0001
FADOI-COMPLIMED Score 2	1.23	(1.10–1.38)	0.0003
1-year Mortality			
FADOI-COMPLIMED Score 1	1.25	(1.18–1.33)	< .0001
FADOI-COMPLIMED Score 2	1.27	(1.14–1.42)	< .0001

### 3.3 Comparison between FADOI-COMPLIMED score(s) and MPI, and internal validation

**Fig 3:**
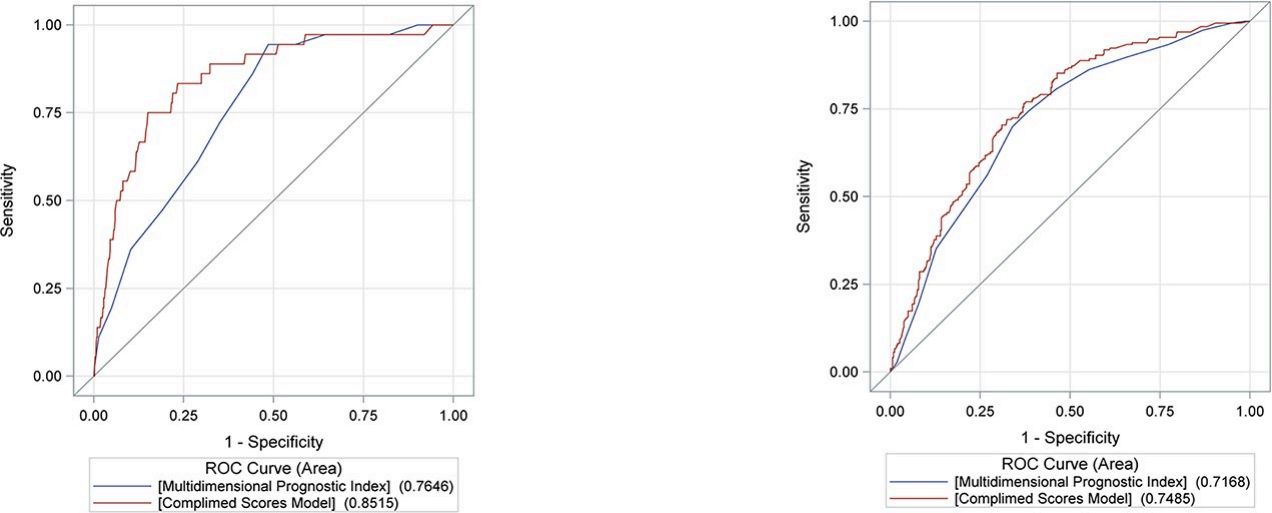
ROC curves for the MPI and FADOI-COMPLIMED score(s) predicting in-hospital mortality (Fig 3a) and 1-year mortality (Fig 3b), respectively.

The predictive ability of FADOI-COMPLIMED Score(s) and the reference MPI index were determined by means of ROC-curve analyses (see Fig 3 with sub-figures for in-hospital and 1-year mortality, the comparisons for 1-month, 3-month and 6-month mortality are provided in the Supplementary Materials) with relevant results (ROC-curve areas and differences) reported in Table 3. To note, the logistic model based on FADOI-COMPLIMED Score(s) is significantly more predictive than the reference and validated model based on MPI for all the time-points considered.

**Table 3:** Comparison of prognostic models based on FADOI-COMPLIMED score(s) and MPI by means of ROC curves.

Clinical Event	ROC Curve Area		Difference with 95% CI	P-Value
	FADOI-COMPLIMED Score(s)	Multidimensional Prognostic Index		
In-hospital Mortality	0.8515	0.7646	0.09 (0.04–0.13)	0.0003
1-month Mortality	0.8033	0.7501	0.05 (0.02–0.09)	0.0055
3-month Mortality	0.7757	0.7393	0.04 (0.01–0.07)	0.0190
6-month Mortality	0.7702	0.7312	0.04 (0.01–0.07)	0.0077
1-year Mortality	0.7485	0.7168	0.03 (0.01–0.06)	0.0313

Since we cannot exclude that the different number of independent variables (risk factors) across the different predictive models may have introduced some optimism in the computation and comparisons of the C-Indices (the higher the number of covariates, the higher the risk of overestimation of the predictive ability), an internal validation based on bootstrap resampling was carried out in order to obtain bias-corrected estimates of the C-Index which should make the predictive models fully comparable to each other. This validation confirms the true higher predictiveness of FADOI-COMPLIMED Score(s) when compared with MPI (S1 Table).

## 4. Discussion

In this paper we have shown that the recently developed and user-friendly FADOI-COMPLIMED Score(s) is a tool with very good predictive power for mortality in patients hospitalized in medical wards, and significantly better than the reference and validated Multidimensional Prognostic Index. In this regard it is worth noting that we did not develop yet another alternative score starting from scratch, but we have limited ourselves to optimizing and using more efficiently the information gathered by internists and geriatricians through questionnaires already available and validated.

Prognosis, like diagnosis and treatment, is one of the major responsibilities and challenges of physicians, and it is becoming increasingly important to clinicians and policy makers for medical decisions [19,20]. Assessment of prognosis may improve the accuracy of the assumptions that influence diagnostic and therapeutic strategies. Indices like the one we propose offer a potential role for moving beyond arbitrary age-based cut-offs in clinical decision-making [1,21], and may be useful in identifying both high- and low-risk patients so that specific interventions can be targeted to each category. This objective is particular challenging and relevant for older people, who have a great diversity of chronic conditions, functional limitations and social challenges (“complexity”) that impact health, quality of life, and the benefits and risks of medical interventions [22]. In view of this heterogeneity, our efforts were aimed at developing an index that was representative of the complexity of patients, and we chose to compare its predictive value with a previously validated instrument, the MPI, based on comprehensive geriatric assessment. The MPI is a reliable and sensitive measurement of risk assessment, and the even more accurate predictive result we obtained with the FADOI-COMPLIMED Score(s), which combines information related to the domains of functional dependence/frailty and comorbidity, appears to confirm that a multidimensional approach may effectively estimate life expectancy and likely performs better than models that consider comorbidity or functional status only [19].

As an outcome to assess the predictive value of the index, in our study we focused on mortality. This choice seems to us reasonable and justified since it is dichotomous and unequivocal, and considering that the predictive capacity of available scores has mainly been studied for mortality, while evidence on other outcomes in the elderly population, such as cardiovascular or cancer events, is limited [23]. Unlike other scores that have documented their predictive ability at fixed and univocal times (e.g. 1 year) [6, 11, 14, 16], a risk function based on FADOI-COMPLIMED Scores shows a sustained prognostic efficiency at different time points (during hospital stay, and after 1-3-6 and 12 months). This may be of particular interest since short-term mortality in patients hospitalized in medical wards is not negligible, and an appropriate stratification of risk could support patient management during hospital stay and early after discharge.

There is a substantial difference between our approach and that normally used in the development of the vast majority of scores, including the MPI itself. Indeed, the latter are usually obtained directly from the estimates of a prognostic model (for example a multivariable logistic regression) specific for a given event detected in a given time with the consequence that the power to predict events is expected to be good especially, if not only, towards the clinical outcome which was used to estimate them. Conversely, our objective was not directly targeted to the formulation of a generic index predictor of mortality or other specific clinical events, but rather to achieving a tool that was able to measure the level of complexity. This is why the FADOI-COMPLIMED Score(s) should be considered as a “generalist tool” which could be used to profitably predict all the complexity-related endpoints, and be valid at different time-points. Moreover, since complexity is a key factor in predicting mortality in an elderly population, it is likely to expect a strong link between the degree of complexity measured by FADOI-COMPLIMED Score(s) and the associated probability of death.

In our cohort the FADOI-COMPLIMED Score(s) outperforms the MPI as predictor of mortality at different time-points, and this result was obtained despite the fact that FADOI-COMPLIMED requires a number of items that is almost half of MPI (34 vs 63). This finding has to be weighted with the fact that MPI was designed as a predictive tool for 1-year mortality, and may be at odds with previous studies suggesting that development of prognostic indices based on a limited number of health problems should be considered with caution [24,25]. However, the rigorous selection we carried out through the two-step strategy described elsewhere [15] plausibly led to the effective identification of items with higher informative and discriminative power, while limiting the inclusion of redundant information. Furthermore, our previous analyses have clearly identified functional dependence/frailty and comorbidity as the two main independent domains of complexity; it follows that a bivariable model, which assesses the independent contribution of each single domain to the risk of death, appears more appropriate to estimate the strength of complexity in predicting mortality than a prognostic tool based on a single index only. This statement is corroborated by the results that emerged in our study which show how the predictive power for mortality due to the dependence/frailty domain increases or decreases compared to that of co-morbidity depending on the detection time. In particular, the importance of the first component of complexity appears to be predominant in predicting short- and medium-term mortality, unlike comorbidity that seems to play a more relevant role in long-term mortality. These findings could be of interest and worth addressing for future research.

Our prognostic model may have some limitations. Since it was developed in hospitalized medical patients, it is likely that it will not be applicable to community-dwelling older adults, nor to other institutional populations. Furthermore, our follow-up and therefore assessment of prognosis was limited to one year. However, the clinical setting we evaluated is probably of great interest since our study population is included in the category of patients with multiple chronic complex diseases who account for a very relevant percentage of all hospital stays, and are considered a priority by many healthcare organizations [14]. Moreover, people with chronic, progressive and disabling illnesses often require hospitalization during the advanced stages, leading to further functional decline and morbidity [26,27]. Hospitalization therefore may become a key trigger point for identifying persons at greatest risk for mortality in the ensuing year. Finally, our study design included a follow-up for survival and major outcomes at 1-3-6 months, and we were therefore able to estimate the prognostic value of our indices at those time points as well.

In conclusion, in this paper we propose a new, efficient and easy-to-use instrument based on FADOI-COMPLIMED Score(s), which demonstrated a good predictive power for mortality in patients hospitalized in medical wards. This tool may be of interest for clinical practice, since it well balances feasibility (requiring the compilation of 34 items, which take around 10 minutes) and performance. Future research should focus on additional validation of this prognostic model, on prospectively testing its validity across diverse clinical settings, and ideally analyzing its impact on clinical decision-making and patient outcomes.

## Supporting information

S1 Fig Surface plot showing the probability of 1-month mortality as a function of COMPLIMED score(s). (DOC)

S2 Fig Surface plot showing the probability of 3-month mortality as a function of COMPLIMED score(s). (DOCX)

S3 Fig Surface plot showing the probability of 6-month mortality as a function of COMPLIMED score(s). (DOCX)

S4 Fig ROC curves for the MPI and COMPLIMED score(s) predicting 1-month mortality. (DOCX)

S5 Fig ROC curves for the MPI and COMPLIMED score(s) predicting 3-month mortality. (DOCX)

S6 Fig ROC curves for the MPI and COMPLIMED score(s) predicting 6-month mortality. (DOCX)

S1 Table Results of internal validation for the examined predictive models. (DOCX)
