# Supplementary material for: Prognostic value for mortality of the new FADOI-COMPLIMED score(s) in patients hospitalized in medical wards
Source: PLoS One. 2019 Jul 24;14(7):e0219767. doi: 10.1371/journal.pone.0219767 (PMC6656348; doi:10.1371/journal.pone.0219767)
Supplement: S1 Fig — (DOC) [file pone.0219767.s001.doc]

**S1 Fig.**

**Surface Plot showing the probability of 1-month mortality as a function of COMPLIMED Score(s)**

**
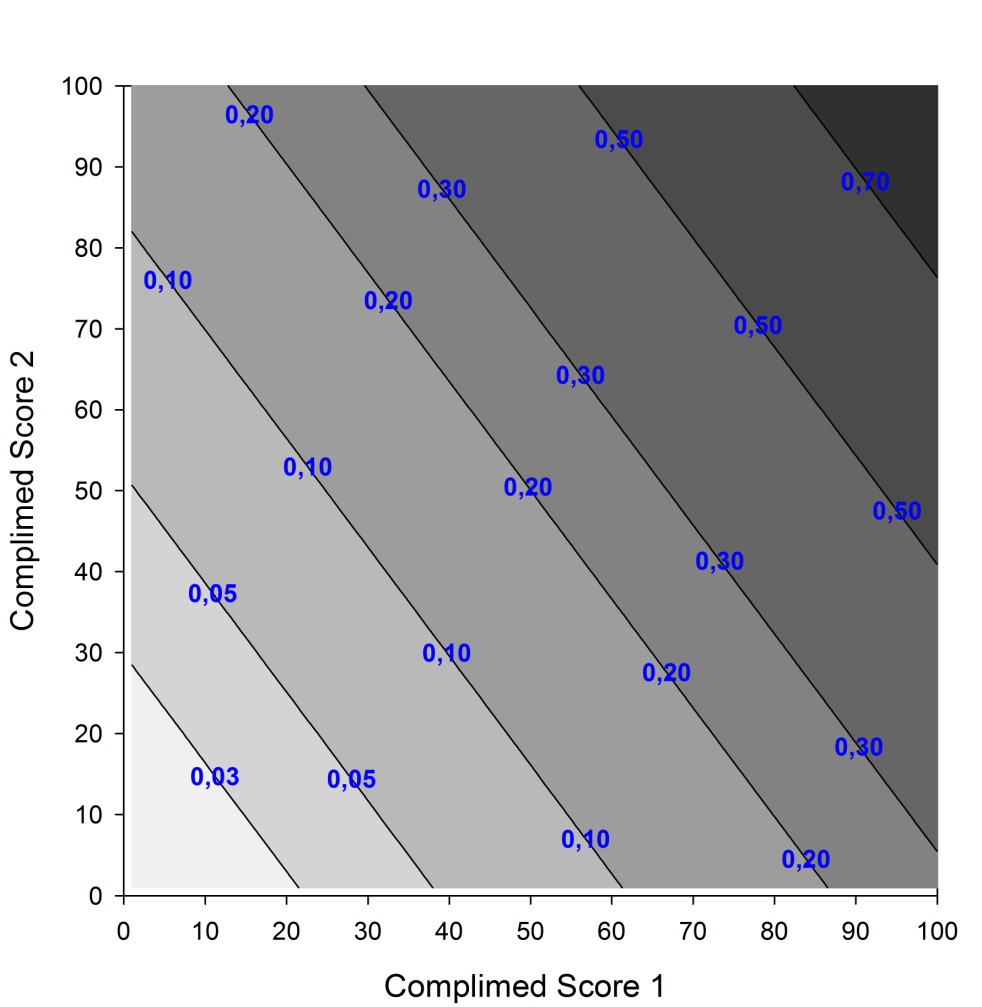
**
