# Supplementary material for: Prognostic value for mortality of the new FADOI-COMPLIMED score(s) in patients hospitalized in medical wards
Source: PLoS One. 2019 Jul 24;14(7):e0219767. doi: 10.1371/journal.pone.0219767 (PMC6656348; doi:10.1371/journal.pone.0219767)
Supplement: S2 Fig — (DOCX) [file pone.0219767.s002.docx]

**S2 Fig.**

**Surface Plot showing the probability of 3-month mortality as a function of COMPLIMED Score(s)**

**
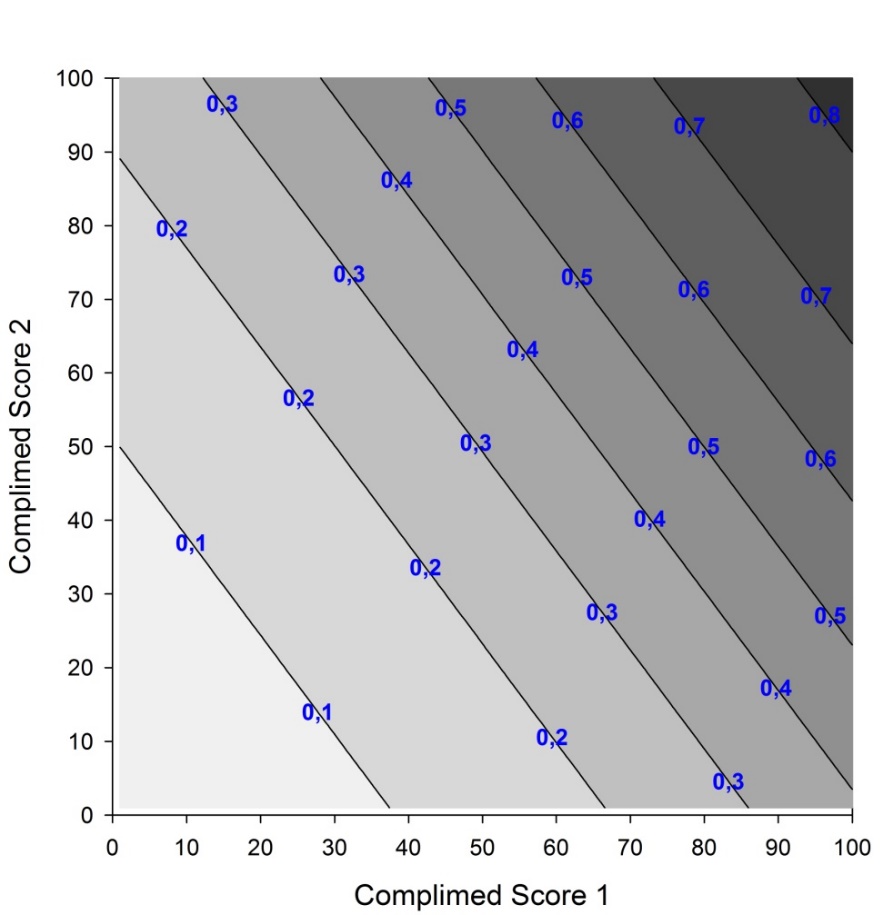
**
