# Supplementary figures and images for: Prognostic value for mortality of the new FADOI-COMPLIMED score(s) in patients hospitalized in medical wards
Source: PLoS One. 2019 Jul 24;14(7):e0219767. doi: 10.1371/journal.pone.0219767 (PMC6656348; doi:10.1371/journal.pone.0219767)

**S4 Fig.**

**ROC curves for the MPI and COMPLIMED Score(s) predicting 1-month mortality**

**
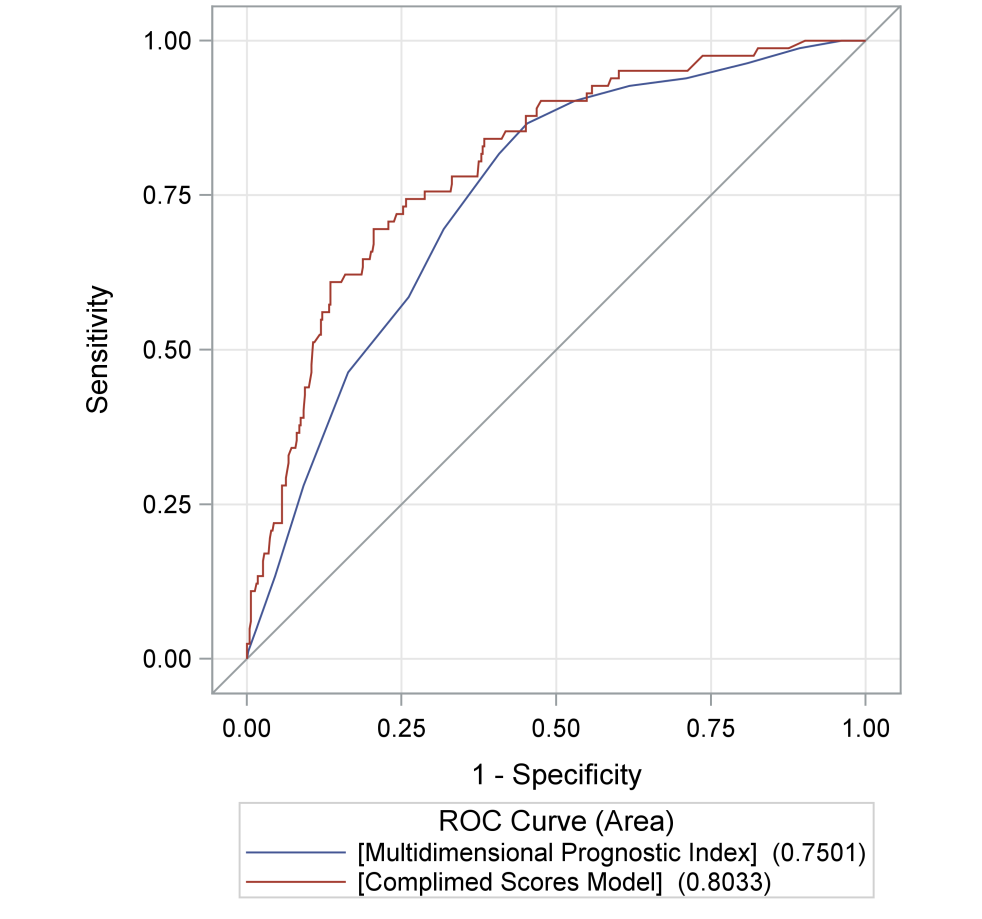
**

Supplement: S4 Fig — (DOCX) [file pone.0219767.s004.docx]

**S5 Fig.**

**ROC curves for the MPI and COMPLIMED Score(s) predicting 3-month mortality**

**
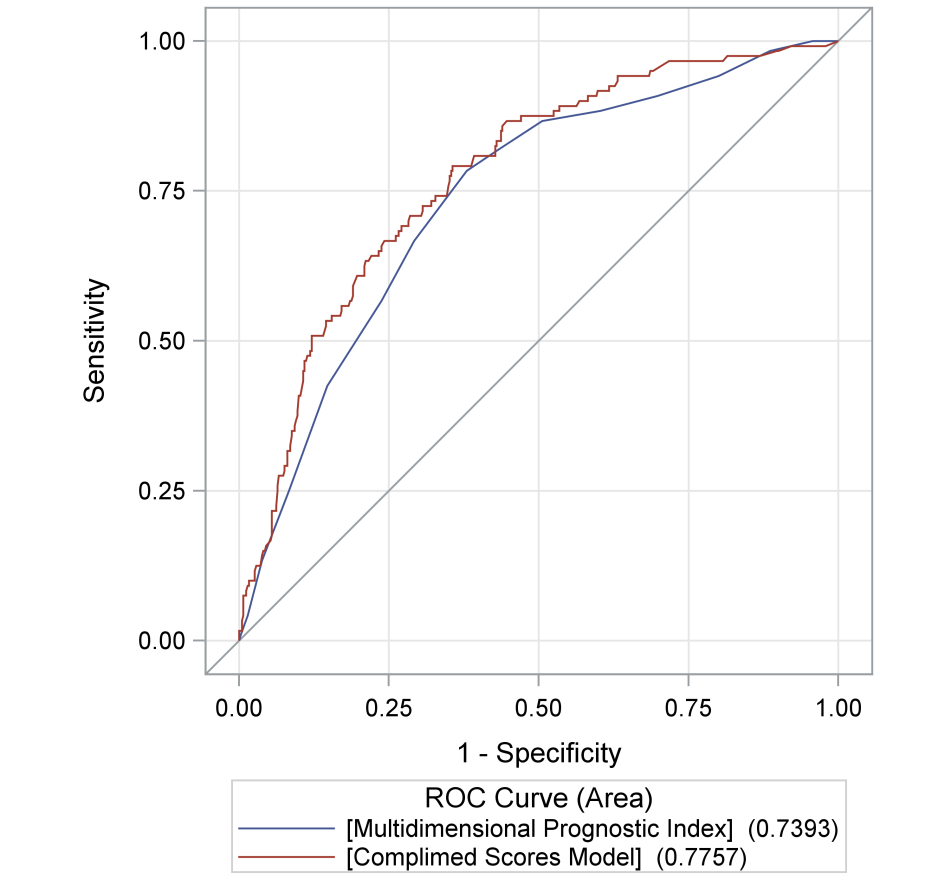
**

Supplement: S5 Fig — (DOCX) [file pone.0219767.s005.docx]

**S6 Fig.**

**ROC curves for the MPI and COMPLIMED Score(s) predicting 6-month mortality**

**
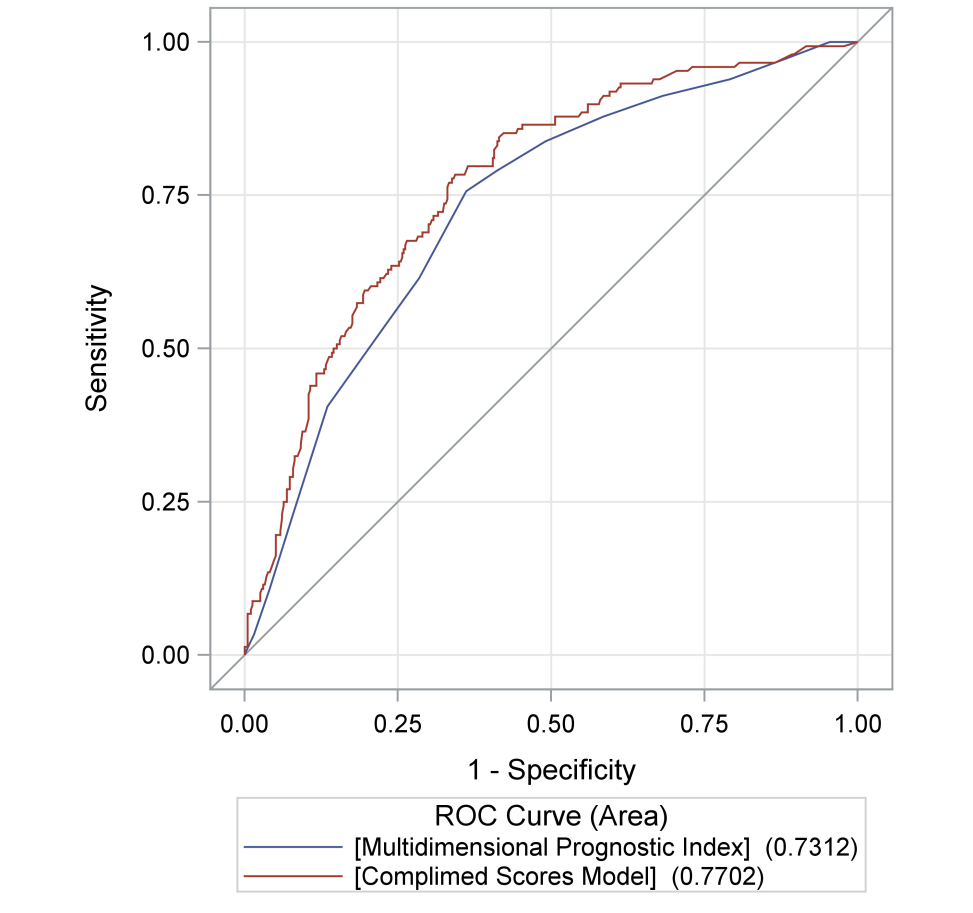
**

Supplement: S6 Fig — (DOCX) [file pone.0219767.s006.docx]
