# Supplementary material for: Prognostic value for mortality of the new FADOI-COMPLIMED score(s) in patients hospitalized in medical wards
Source: PLoS One. 2019 Jul 24;14(7):e0219767. doi: 10.1371/journal.pone.0219767 (PMC6656348; doi:10.1371/journal.pone.0219767)
Supplement: S1 Table — (DOCX) [file pone.0219767.s007.docx]

**S1 Table. Results of Internal Validation for the examined predictive models**

| **Clinical Event / Predictive Model** | **Original**  **C-Index (A)** | **Training**  **C-Index (B)** | **Test**  **C-Index (C)** | **Optimism Bias (D)** | **Bias-Corrected**  **C-Index (E)** |
| --- | --- | --- | --- | --- | --- |
| In-hospital Mortality |  |  |  |  |  |
| Multidimensional Prognostic Index | 0.7646 | 0.7645 | 0.7646 | -0.0001 | 0.7647 |
| FADOI-COMPLIMED Score(s) | 0.8515 | 0.8522 | 0.8496 | 0.0027 | 0.8488 |
| 1-month Mortality |  |  |  |  |  |
| Multidimensional Prognostic Index | 0.7501 | 0.7512 | 0.7501 | 0.0011 | 0.7491 |
| FADOI-COMPLIMED Score(s) | 0.8033 | 0.8047 | 0.8020 | 0.0026 | 0.8006 |
| 3-month Mortality |  |  |  |  |  |
| Multidimensional Prognostic Index | 0.7393 | 0.7398 | 0.7393 | 0.0005 | 0.7388 |
| FADOI-COMPLIMED Score(s) | 0.7757 | 0.7756 | 0.7746 | 0.0010 | 0.7747 |
| 6-month Mortality |  |  |  |  |  |
| Multidimensional Prognostic Index | 0.7312 | 0.7311 | 0.7312 | -0.0001 | 0.7313 |
| FADOI-COMPLIMED Score(s) | 0.7702 | 0.7701 | 0.7690 | 0.0011 | 0.7691 |
| 1-year Mortality |  |  |  |  |  |
| Multidimensional Prognostic Index | 0.7168 | 0.7174 | 0.7168 | 0.0006 | 0.7162 |
| FADOI-COMPLIMED Score(s) | 0.7485 | 0.7497 | 0.7476 | 0.0021 | 0.7464 |

1. C-Index computed from the original data. These estimates correspond to the areas under the ROC curves depicted in Figure 3, and were the basis for the statistical comparisons reported in Table 3.
2. Average of C-Indexes estimated from 1000 bootstrap training samples.
3. Average of C-Indexes obtained by evaluating the 1000 bootstrap logistic model estimates on the original data.
4. Bias which occurs when a predictive model is used to predict the same events previously employed to build the model itself. It is computed from the difference between B and C.
5. C-Index computed by subtracting the optimism from the Original C-index. The Bias-Corrected C-Index should estimate the “true” ability of a risk function to predict new events.
